# *In vivo *imaging of pancreatic tumours and liver metastases using 7 Tesla MRI in a murine orthotopic pancreatic cancer model and a liver metastases model

**DOI:** 10.1186/1471-2407-11-40

**Published:** 2011-01-28

**Authors:** Ivo L Partecke, André Kaeding, Matthias Sendler, Nele Albers, Jens-P Kühn, Sven Speerforck, Sebastian Roese, Florian Seubert, Stephan Diedrich, Sandra Kuehn, Ulrich F Weiss, Julia Mayerle, Markus M Lerch, Stefan Hadlich, Norbert Hosten, Claus-D Heidecke, Ralf Puls, Wolfram von Bernstorff

**Affiliations:** 1 Department of General, Visceral, Thoracic and Vascular Surgery, Ernst-Moritz-Arndt-University, Friedrich-Loeffler-Str. 23 b, Greifswald, Germany; 2 Department of Medicine A, Ernst-Moritz-Arndt-University, Friedrich-Loeffer-Str. 23a, Greifswald, Germany; 3 Department of Radiology, Ernst-Moritz-Arndt-University, Ferdinand-Sauerbruchstraße Greifswald, Germany

## Abstract

**Background:**

Pancreatic cancer is the fourth leading cause of tumour death in the western world. However, appropriate tumour models are scarce. Here we present a syngeneic murine pancreatic cancer model using 7 Tesla MRI and evaluate its clinical relevance and applicability.

**Methods:**

6606PDA murine pancreatic cancer cells were orthotopically injected into the pancreatic head. Liver metastases were induced through splenic injection. Animals were analyzed by MRI three and five weeks following injection. Tumours were detected using T2-weighted high resolution sequences. Tumour volumes were determined by callipers and MRI. Liver metastases were analyzed using gadolinium-EOB-DTPA and T1-weighted 3D-Flash sequences. Tumour blood flow was measured using low molecular gadobutrol and high molecular gadolinium-DTPA.

**Results:**

MRI handling and applicability was similar to human systems, resolution as low as 0.1 mm. After 5 weeks tumour volumes differed significantly (p < 0.01) when comparing calliper measurments (n = 5, mean 1065 mm^3^+/-243 mm^3^) with MRI (mean 918 mm^3^+/-193 mm^3^) with MRI being more precise. Histology (n = 5) confirmed MRI tumour measurements (mean size MRI 38.5 mm^2^+/-22.8 mm^2 ^versus 32.6 mm^2^+/-22.6 mm^2 ^(histology), p < 0,0004) with differences due to fixation and processing of specimens. After splenic injection all mice developed liver metastases with a mean of 8 metastases and a mean volume of 173.8 mm^3^+/-56.7 mm^3 ^after 5 weeks. Lymphnodes were also easily identified. Tumour accumulation of gadobutrol was significantly (p < 0.05) higher than gadolinium-DTPA. All imaging experiments could be done repeatedly to comply with the 3R-principle thus reducing the number of experimental animals.

**Conclusions:**

This model permits monitoring of tumour growth and metastasis formation in longitudinal non-invasive high-resolution MR studies including using contrast agents comparable to human pancreatic cancer. This multidisciplinary environment enables radiologists, surgeons and physicians to further improve translational research and therapies of pancreatic cancer.

## Background

Pancreatic cancer is the third most common cause of gastrointestinal malignancies and the fourth leading cause of cancer death in the northern hemisphere. Pancreatic cancer accounts for approximately 33,000 cancer deaths in the United States and almost 13,000 in Germany [1-3]. Despite significant improvements in surgical and non-surgical treatment modalities including new possible therapeutic targets [4,5] the almost unchanged 5-year survival rate of 5% remains poor. This is also mirrored by the general lack of effective medical treatment options [6]. Therefore, reproducible preclinical models are required to study the underlying causes of tumour development, growth and dissemination of pancreatic cancer. Also, these models are crucial for the development of new and effective treatment modalities as well as the evaluation of diagnostic imaging techniques for monitoring tumour growth and metastasis formation.

Currently, several mouse models of pancreatic cancer have been established [7]. These include subcutaneously [8,9] and/or orthotopically [10,11] implanted xenografts of human tumour cells into SCID or nude mice i.e. using mice with defects of their immune system. Even lung metastases may be generated with these models [8,9]. Other models include the orthotopic injection or transplantation of established syngeneic pancreatic mouse cancer cell lines like Panc02 [12,13] or 6606PDA [14] into the appropriate immuno-competent mouse strain. Further research has succeeded in the development of new syngeneic orthotopic models as recently published by Tseng et al. [15].

The aim of this study was the evaluation of clinically relevant murine pancreatic cancer models for longitudinal diagnostic and therapeutic studies. An orthotopic pancreatic cancer model as well as a liver metastases model using the cell line 6606PDA [14] were studied using a 7 Tesla magnetic resonance imager. The monitoring of tumour growth and the formation of metastases was examined focussing on the possibility of reducing the number of animals needed for long term follow-up according to the 3R-principles (reduction, replacement and refinement of research animals) described by Russel and Burch 1959 [16]. The complete system mimics the human clinical situation including the cooperation of medical, surgical and radiological specialists.

## Methods

### Laboratory Animals

Six to eight weeks old male C57/BL6 mice with a body weight of 20 to 23 g were obtained from Charles River Laboratories (Bad Sulzfeld, Germany) and allowed to adapt to the new surrounding for about seven to fourteen days. They were maintained in a specific pathogen-free environment receiving sterilized food (ssniV R-Z, ssniV Spezialdiäten GmbH, Soest, Germany) and tap water ad libitum. Stress levels were kept to a minimum before starting experiments. Animal rooms had a twelve to twelve hour light-dark/day-night cycle and were maintained at constant temperature and humidity. Before starting experiments all animal studies had been approved by the ethics committee for animal care of Mecklenburg-Vorpommern, Germany.

### Cell Line and Culture

The murine pancreatic adenocarcinoma cell line 6606PDA was a kind gift from Prof. David Tuveson, Cambridge, UK [14]. 6606PDA cells have been isolated from a pancreatic adenocarcinoma of a transgenic C57/BL6 mouse carrying a Kras^D12G ^allele. Cells were maintained in RPMI-1640 medium supplemented with 10% fetal calf serum, 100 U/ml of penicillin and 100 μg/ml of streptomycin (referred to as "complete medium"). Tissue culture reagents were obtained from Gibco (Invitrogen, Carlsbad, California, USA). Cell cultures were kept in a humidified incubator at 37°C with 5% CO_2_. Cell cultures were kept pathogen-free and were regularly tested for *Mycoplasma *species. They were consistently negative for *Mycoplasma *contamination.

### Orthotopic Injection Technique

Subconfluent cultures of 6606PDA cells were harvested using a 0.05% Trypsin-solution, washed twice in PBS, and re-suspended as single-cell suspensions in serum-free RPMI-1640. Cell viability was tested using trypan blue exclusion. Viability was always greater than 95%.

General anaesthesia in mice was induced using a combination of ketamine hydrochloride (Ketanest S^® ^Pfizer Pharma, Berlin, Germany) and xylometazoline hydrochloride (Rompun^® ^Bayer HealthCare, Berlin, Germany) at concentrations of 87 mg/kg and 13 mg/kg injected intraperitoneally. The abdominal cavity was opened by a 1.5 cm wide transverse laparotomy pointing slightly to the right. The head of the pancreas was identified and lifted up by a cotton wool tip. 2.5 × 10^5 ^tumour cells in a total volume of 20 μl consisting of equal volumes of PBS and matrigel (Matrigel™ Basement Membrane Matrix, BD Bioscience, San José, CA, USA) were slowly injected into the head of the pancreas using a 27-gauge needle. Matrigel is a substance with a more watery consistency at 4°C but turns into a gel-like substance when reaching temperatures above 22°C. It was used to prevent local spillage and thereby the early development of local peritoneal tumour growth. To further prevent leakage a cotton wool tip was pressed onto the injection site for 30 seconds. To ensure the same injection technique for all experiments the injection was always carried out by the same person (M.S.). The pancreas was then replaced into the abdominal cavity. The abdominal cavity was closed by a running single layer 4/0 polyester suture (Catgut, Markneukirchen, Germany). To decrease post surgical pain and the effects of surgery we subcutaneously injected buprenorphine hydrochloride at a dose of 0,1 mg/kg. So far, we have not had any fatalities related to surgery.

### Induction of liver metastases by splenic injection

General anaesthesia in mice was induced as above. The abdominal cavity was opened by a 0.5 cm left sided transverse laparotomy. The spleen was identified and lifted up by a cotton wool tip. 5 × 10^5 ^6606PDA cells in a total volume of 50 μl of PBS were injected subcapsularly into the spleen using a 27 gauge needle. To prevent leakage a cotton wool tip was pressed onto the injection site for 30 seconds. The closure of the abdominal cavity and post surgical pain therapy were carried out as above. Following this splenic injection technique we have not had any surgery related fatalities either.

### 7 Tesla MRI

**Figure 1:**
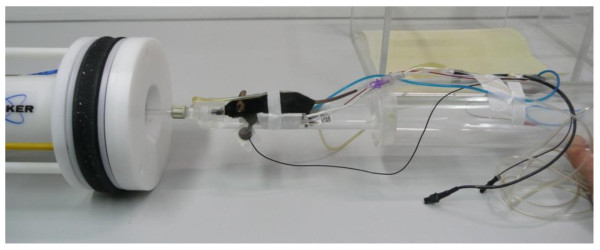
**MRI equipment**. Mouse prepared for MRI scan monitoring the vital parameters breathing rate, heart rate and temperature, position in front of coil

For all MRI studies anaesthesia had to be carried out using isoflurane (1% - 1.5%). The depth of anaesthesia was monitored by the breathing rate. MRI sequences were triggered by the breathing rate. An anaesthesia adjusted breathing rate of 40 per minute was found to be optimal. To reduce the influence of bowel motility in all MRI examinations mice were always kept *nil per os *(NPO) for at least 4 hours before starting MRI scans. For MRI scans all mice were kept in a supine position (Figure 1). For all MRI studies mice were scanned after 3 and after 5 weeks of tumour injection. However, other intervals and more frequent scans can be done without any obvious harm to the mice.

**Figure 2:**
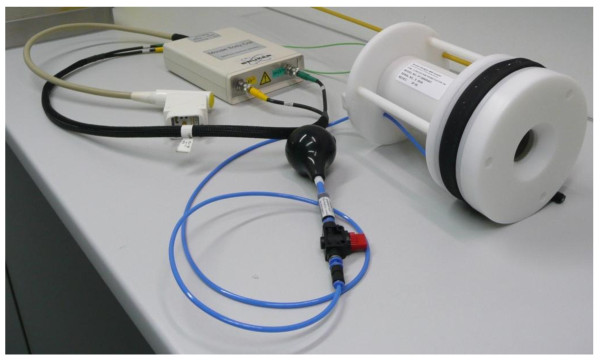
**Whole body mouse coil**.

Tumour bearing mice were scanned in a high field 7.0 Tesla MRI scanner for small animals (Bruker, ClinScan, 7.0 Tesla, 290 mTesla/m gradient strength (Bruker, Ettlingen, Germany)). MRI analyses were performed in a whole mouse body coil (Figure 2, Bruker, Ettlingen, Germany) using a T2-TSE (turbo spin echo) sequence. The whole body coil is a volume coil that allows the imaging of the whole mouse without inducing any inhomogeneities of the signal. For the assessment of tumour sizes we used high resolution T2-weighted images of the frontal as well as the horizontal plane (frontal plane: TR (repetition time): ca. 1200 ms; TE (echo time): 41.0 ms; FA (flip angle): 180°; FoV (field of view): 42 mm × 42 mm; matrix: 240 × 320; 24 slices of 0,7 mm per slice, acquisition time: ca. 15 min; horizontal plane: TR: ca. 1250 ms; TE: 41.0 ms; FA: 180°; FoV: 40 mm × 40 mm; matrix: 240 × 320; 24 slices of 0.7 mm per slice, acquisition time: ca. 10 min). Generated images were analyzed employing MIPAV (medical imaging processing and visualisation, National Institutes of Health, Bethesda, Maryland, USA). All image slices of the tumours were marked with so called regions of interest (ROI) which facilitated the software calculation of the tumour volume. This was finally done with a complex algorythm using all image inherent information including thickness of slices, resolution as well as size of ROIs.

The definition of ROI was carried out in a reproducible and standardized way by one investigator only (AK) applied to all mice. However, difficult findings or images were always discussed with a senior radiologist. The investigator had been thoroughly trained by analyzing numerous MR scans and optimized the MR analyses for the presented murine models in cooperation with the involved radiologists. Satellite tumours were considered as part of the original tumour if there was true and continuous tumour between original tumour and satellite tumour. Metastases and secondary tumours without continuous tumour growth connecting to the original tumour were excluded when measuring the primary tumour. The tumour borders/edges could easily be identified even without contrast media due to their characteristic shape and appearance. MR images were compared with corresponding macroscopic tumour growth on exploration and with corresponding microscopic tumour growth using histological specimens.

To analyze the acquired MRI data we used CIT (Center for Information Technology) and NIH (National Institute of Health) developed algorithms in MIPAV-software (Medical Image Processing and Visualization [17]). To validate the used volumetric algorithms for compatibility with our MRI-system we examined 1000 mm^3 ^water of defined volume (calibrated pipette) in a standardized 1.5 ml Eppendorf tube as an MRI phantom.

### Imaging of liver metastases

For the generation of images of liver metastases 200 μl of the contrast medium Gadolinium ethoxybenzyl diethylenetriamine pentaacetic acid (Gd-EOB-DTPA; gadoxetic acid disodium, Primovist^®^, Bayer Schering Pharma, Berlin, Germany) were injected intravenously 10 minutes before starting MRI scans. Primovist^® ^was used at a concentration of 0.025 mmol/ml and was injected via the tail vein. Also for these experiments mice were kept in a supine position using the whole mouse body coil. For the detection of liver metastases a high resolution T1 flash3d-vibe („**v**olumetric **i**nterpolated **b**reathhold **e**xamination") weighted sequence was used. Scans were carried out in the frontal as well as the horizontal plane (frontal and horizontal plane: TR: ca. 10 ms; TE: 1.46 ms; FA: 12°; FoV: 50 mm × 50 mm; matrix: 256 × 256; 48 slices of 0.3 mm each slice; acquisition time: ca. 2:45 min.). This was followed by the generation of high resolution T2-weighted images in frontal and horizontal planes as above. The volumes of liver metastases as well as the volume of the liver itself were calculated using MIPAV using the above described algorithm.

### Dynamic contrast enhanced MRI (DCE-MRI) for the imaging of tumour blood flow

For imaging studies of the blood flow within the tumour two different contrast media were used: the low molecular weight contrast medium gadobutrol (Gadovist^®^, Bayer Schering Pharma, Berlin, Germany) and the high molecular weight contrast medium gadolinium-DTPA (Gadomer^®^, Bayer Schering Pharma, Berlin, Germany). So far, neither of them has induced any adverse reactions.

For imaging studies an intravenous catheter (26G BD Vasculon™ Plus, BD, USA) was inserted into the tail vein of the mouse. The catheter was connected with an injection line and linked to the injection pump (Continuum MR System, Medrad, Indianola, PA, USA). Again, for these experiments mice were kept in a supine position using the whole mouse body coil. For the tumour assessment two fast high resolution T2-weighted scans were done: in the frontal as well as the horizontal plane (frontal plane: TR: ca. 2030 ms; TE: 40.0 ms; FA: 180°; FoV: 33 mm × 33 mm; matrix: 144 × 192; 15 slices at 1.5 mm each; acquisition time: ca. 2:53 min; horizontal plane: TR: 2693 ms; TE: 41.0 ms; FA: 180°; FoV: 40 mm × 40 mm; Matrix: 240 × 320; 5 slices at 2 mm each, acquisition time: ca. 3:11 min).

Thereafter, the FoV was determined. It was always essential to have the centre of the FoV within the largest diameter of the tumour. This was followed by T1-weighted pre-contrast scans (horizontal: TR: 500 ms; TE: 14.6 ms; FA: 180°; FoV: 25 mm × 25 mm; matrix: 320 × 512 interpolated; 20 slices at 0.5 mm per slice; acquisition time: 2:42 min). Several pre-contrast scans were taken for an acquisition of a statistically stable baseline After completing 9 pre-contrast scans the dynamic contrast medium enhanced scans were performed using the VIBE-recording technique (***V****olume ***I**nterpolated **B**reathhold **E**xamination, horizontal plane: TR: ca. 3.98 ms; TE: 1.89 ms; FA: 12°; FoV: 50 mm × 50 mm; matrix: 192 × 256; 8 slices at 1.5 mm each slice, acquisition time: 637 sec, total number of pictures: 130, acquisition time: ca. 13:00 min). The contrast medium was injected via the tail vein. The optimal concentration and amount of the contrast medium was calculated using the weight of the mouse. The standard concentration of the contrast medium was 0.1 mmol/kg body weight, i.e. 0.0025 mmol/ml for a 25 g mouse. For a standardized application of the contrast medium (bolus of 200 μl within 3 seconds) an injection pump with bolus function was used (Continuum MR System, Medrad, Indianola, PA, USA).

To minimize the negative effects of changes in vital parameters of the blood flow, microcirculation and the distribution of the contrast medium the breathing rate, the heart rate and the temperature were monitored closely and kept within narrow limits (Figure 1: breathing rate: 30-50/min.; heart rate: 200-400/min.; temperature: 36,5°C (+/- 0,5°C)).

The basis for measuring tumour blood flow was a model in which the accumulation of contrast medium within the tumour over time was used as the marker. The accumulation was analyzed through the change of signal intensity within a defined ROI. This ROI was placed around the whole tumour. Before injecting the contrast agent/medium the base line of the signal intensity was measured. For this altogether 10 analyses were carried through. The mean of analyses 4 through 9 was taken as the base line SI_0_. Then, the SI after injection of the contrast medium SI_t _was measured. To determine an SI-index mirroring the accumulation of the contrast agent within the tumour (i.e. the actual change of the contrast agent over time) reflecting the tumour blood flow the base line SI_0 _was subtracted from SI_t _divided by the base line value: ΔSI_t _= (SI_t _- SI_0_)/SI_0_

This SI-index ΔSI_t _was measured and calculated for each animal. Finally, the mean SI-index of each group was determined for comparisons between the groups. This method using the above radiological and mathematical means is feasible and reproducible for the exact measurement of tumour blood flow. Furthermore, this method could be used for analyzing different factors that might influence tumour blood flow. The functional distribution of the contrast medium within the tumour tissue was analyzed using the two compartments model of pharmacokinetics [18].

### Calliper measurement and histological examination

Tumour-bearing animals were sacrificed 5 weeks after tumour injection following the final MRI scan. Pancreatic tumours and/or livers were resected. The volume of tumours was estimated by electronic calliper measurements. Tumour volume was calculated using the formula for ellipsoid tumours V = L × W × H × (π/6). L was the longest distance from right to left, W was the largest dorsal/ventral diameter and H was the largest rostral/caudal diameter [19]. Furthermore, histological specimens and MR images were compared for tumour volumes, tumour structure (e.g. necroses), and possible infiltration of surrounding tissues. The number of metastases was estimated by counting them in all recorded image planes. The volume of metastases was determined by MR measurement using the already described methods.

All specimens were analyzed by histology, 5 sections were cut per specimen for routine analysis. Specimens were fixed in 10% buffered formalin, processed routinely, and embedded in paraffin. Tumour rotation during the embedding process was prevented by marking the tumour/pancreatic specimen in situ e.g. by stitches or markers. Then, after fixation, the specimens were transferred into the embedding chambers holding the specimens in position until the paraffin was solid to prevent further rotation. After cutting 4 μm serial sections they were stained using haematoxylin and eosin. Sections were evaluated for various pathologic parameters using a light microscope (Leica, Wetzlar, Germany). Also, the serially cut specimens (20 slides altogether per specimen) were compared with their corresponding MR images. The appropriate matches were found when slide and image showed the same landmarks.

### Statistics

*In vitro *and *in vivo *results were statistically analyzed using the program GraphPad InStat version 3.06 (GraphPad Software Inc., San Diego, California, USA). The unpaired or the paired *t-*test with Welch's correction or the unpaired Mann-Whitney test (for non-Gaussian distributions) or the Wilcoxon signed rank test were taken to compare the means of values of the experiments. A p-value below 0.05 was considered to be statistically significant.

## Results

### Evaluation of tumour size

The validation of the used volumetric-algorithms for the compatibility with our MR-system using Eppendorf tubes showed a mean discrepancy to the expected volume of 1.8% with a calculated mean of all coronal and transverse planes of 982 mm^3 ^(SD +/- 27.2 mm^3^). The one sample t-test (p = 0.36) and the Wilcoxon Signed Rank Test (p = 0.5) revealed no significant (alpha = 0.05) difference to the hypothetical value of 1000 mm^3^.

**Figure 3:**
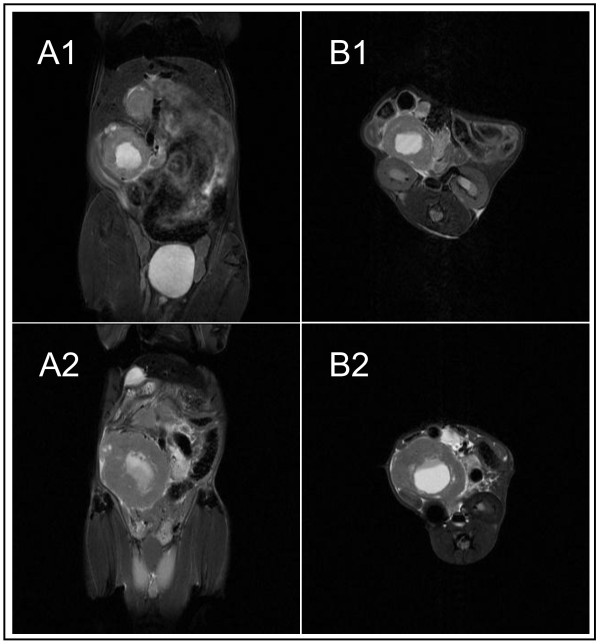
**Monitoring of tumour growth over time**. The orthotopically injected tumour into the head of the pancreas was imaged after 3 und 5 weeks of tumour growth (NB: central necrosis of the tumour): **A**: frontal plane; **A1**: 3 weeks; **A2**: 5 weeks **B**: horizontal plane; **B1**: 3 weeks; **B2**: 5 weeks

**Figure 4:**
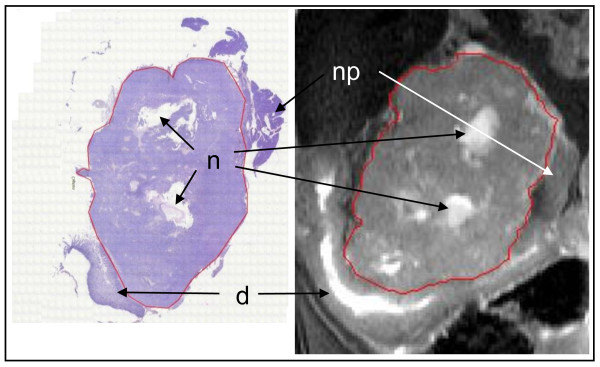
**Monitoring of tumour growth over time**. Comparison of tumour sizes using MRI and histology: Both pictures depict same slice of tumour; right: tumour area equals 66.7 mm^2^, left: 73.1 mm^2^; differences in tumour size (outlined) were due to fixation and always present; yet, slides were well comparable: tumour and surrounding tissues: tumour: within red frame, n: zone of necrosis, np: normal pancreas, d: duodenum

All mice orthotopically injected with tumour cells developed tumours within the head of the pancreas (i.e. 100%). Survival analyses have shown a median survival of about 50 days. To study the size of tumours high resolution T2-weighted images in the frontal as well as the horizontal plane were analyzed. The tumour within the head of the pancreas could be safely identified in all tumour bearing animals. The tumours showed strong signal intensities. With increasing tumour size a typical central zone of tumour necrosis could be identified (Figure 3 and 4).

**Figure 5:**
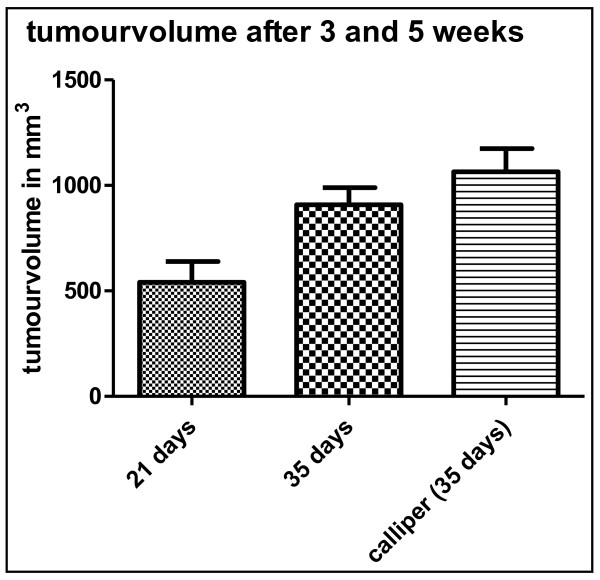
**Monitoring of tumour growth over time**. Tumour volumes after 3 weeks, 5 weeks (MRI) and after 5 weeks by calliper

With the help of breathing rate triggered sequences and the reduction of bowel motility by keeping mice NPO the margins of the tumour could be focused sharply. This was essential for the exact determination of the tumour volume. Tumour growth could be closely monitored over time (n = 5): 21 days following tumour injection the mean tumour volume could be measured at 540 mm^3 ^(range 253 to 835 mm^3^, mean 540 +/- 220 mm^3^). 35 days following tumour injection tumours had increased in volume to 918 mm^3 ^(range 617 to 1118 mm^3^, mean 918 mm^3 ^+/- 193 mm^3^). In addition, tumour size was assessed by using electronic callipers using the formula LxWxHxΠ/6 (see above). Following the last MRI scan after 35 days of tumour growth mice were sacrificed and organs were harvested for further analyses. Tumours measured by callipers were always slightly larger compared to tumours measured by MRI with a mean of 1065 mm^3 ^(range 708 - 1300 mm^3^, +/- 243 mm^3^, p < 0.01, paired t-test compared to MRI tumours, Figure 5). This difference was partly due to surrounding tissues and particularly due to the method of measuring the tumour size using the above formula overestimating the total tumour volumes. This was confirmed in 2 consecutive experiments (n = 8 mice each group) comparing MRI and calliper measurements with expected differences in tumour sizes (395.5 mm^3 ^+/-181.0 mm^3 ^(MRI) versus 498.1 mm^3 ^+/-252.8 mm^3 ^(calliper), p = 0.069; and 316.5 mm^3 ^+/- 318 mm^3 ^(MRI) versus 463.1 mm^3 ^+/-331.9 mm^3 ^(calliper) p < 0.007, paired t-test).

**Figure 6:**
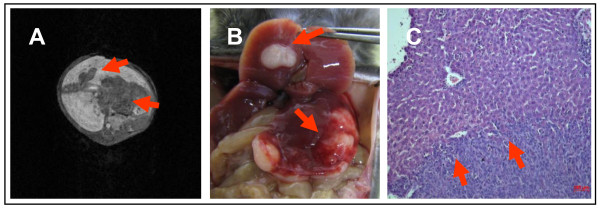
**Liver metastases**. **A**: contrast enhanced images (contrast medium Primovist^®^) 3 weeks after splenic injection: mean liver volume was 1065 mm^3^; mean volume of metastases was 66.9 mm^3^; mean number of metastases was 5.3 **B**: macroscopic findings - arrows pointing at identical sites of MRI and macroscopic specimen **C**: histopathology, magnification 200 x; bar: 100 μm; arrows mark invasive margin at normal liver tissue

**Figure 7:**
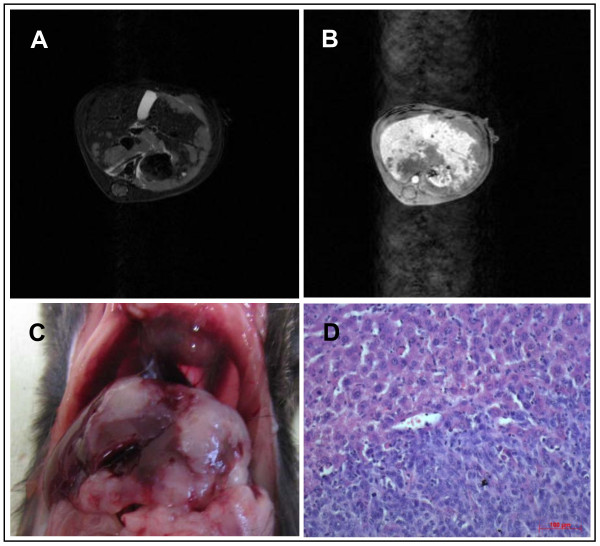
**Liver metastases**. **A**: T2-weighted sequences of liver metastasis **B**: contrast-enhanced image using Primovist^® ^**C**: macroscopic findings **D**: histopathology, magnification 200 ×; bar: 100 μm

**Figure 8:**
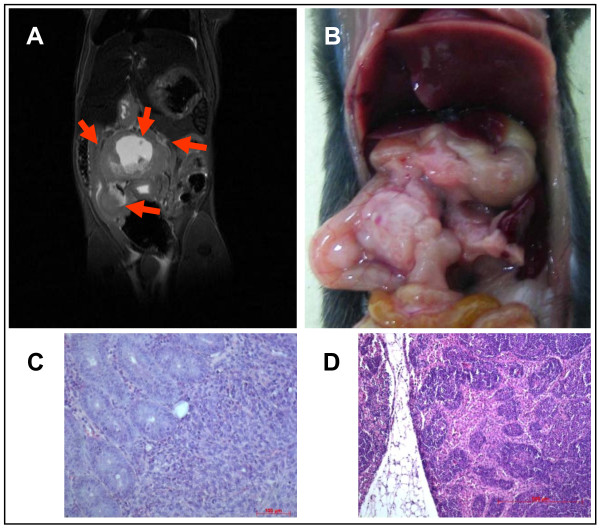
**Invasive tumour and lymphnode metastases**. **A**: local tumour growth after 3 weeks, MRI findings **B**: macroscopic findings; arrows pointing at identical areas of MRI **C**: histopathology shows tumour invasion of small intestine **D**: histopathology shows lymphnode metastasis

Comparing MRI and histology (n = 5 mice, histological slides = 20 for each specimen) there were only small though significant but expected differences in tumour sizes albeit standard deviations were the same. Tumour sizes were compared by using similar sections of MR images and specimen sections the size being measured and compared after electronic scanning (Figure 4, mean size MRI tumours 38.5 mm^2 ^+/-22.8 mm^2 ^versus 32.6 mm^2 ^+/- 22.6 mm^2 ^histological tumours, p < 0,0004, paired t-test). This difference was due to tumour shrinking following fixation and possibly alterations after cutting, staining and coverslipping procedures of tumours and was a systematic effect. Therefore, these differences were constantly present and did not differ significantly between animals. Also, the standard deviation was almost exactly the same, pointing out the systematic effect. Furthermore, the very high spatial resolution of the 7 Tesla MRI enabled the investigator to detect changes as small as 0.1 mm. Therefore, tumour infiltration e.g. of the bowel could be seen on MR images or MR changes were highly suggestive of tumour infiltration. This was confirmed by histological analyses (Figure 3, 4, 6, 7, and 8). In summary, MRI proved to be the most reliable and reproducible method for the measurement of tumour volume and tumour size. Furthermore, the initial injected volume of 20 μl (20 mm^3^) would have been easily detected by MRI. Yet, MRI immediately after injection would have caused too much stress to the mice. The earliest time point of MR imaging was 10 days following injection showing mean tumour volumes of 320 mm^3 ^(range 144 to 606 mm^3 ^+/- 187 mm^3^, n = 6). Matrigel has not shown any effects on tumour growth or MR imaging. Previous in vitro experiments using matrigel and without matrigel had shown almost similar growth and migration of tumour cells. This was confirmed in *in vivo *experiments (data not shown). Lymphnode metastases were easily identified ranging from 0 to 8 (mean 4.5, n = 5 mice) after 5 weeks in this tumour model (data not shown). They presented as round hyper intense lesions measuring up to 5.0 mm in diameter (Figure 8).

### Imaging of liver metastases

**Figure 9:**
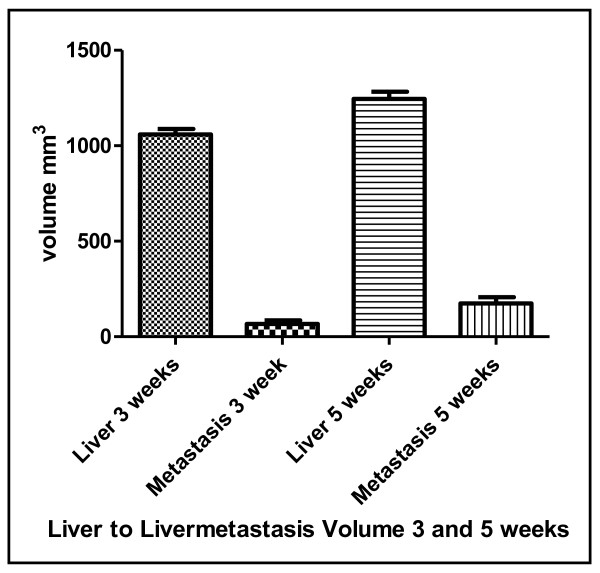
**Liver metastases**. Liver volume and volume of liver metastases after 3 and 5 weeks

Following splenic injection all animals (i.e. 100%, n = 5) developed liver metastases. First, small tumours developed within the spleen before metastasizing via the splenic and the portal vein into the liver (data not shown). After three weeks liver metastases could be detected by MRI. The T1 weighted 3D-Flash sequences proved to be an excellent technique for a three dimensional picture of all anatomical structures. This was accomplished within a short overall scanning time. Primovist^® ^as a liver specific contrast medium imaged the liver hyper intense and liver metastases as negatively contrast enhanced lesions within the liver (Figure 6 and 7). The longitudinal change in number and volume of metastases could be analyzed with the help of MRI specific software. After three weeks an average of 5.3 metastases (range 3 - 8) with a mean volume of 66.9 mm^3 ^(range 40.3 - 103.4 mm^3 ^+/-32.8 mm^3^) was detected. After five weeks these had significantly (p < 0.03) increased to an average of 8 metastases (range 5 - 12) with a mean volume of 173.8 mm^3 ^(range 123.6 - 235.3 mm^3 ^+/-56.7 mm^3^) After measuring the total volume of the liver the percentage of the liver volume that was taken up by the metastases could be determined (Figure 9).

### Dynamic contrast enhanced MRI (DCE-MRI) for the imaging of tumour blood flow

**Figure 10:**
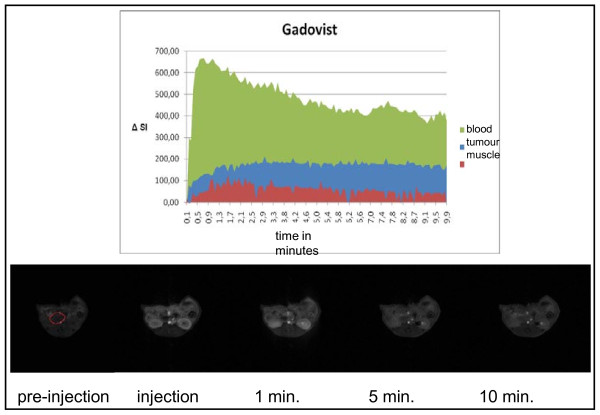
**Tumour blood flow analyses**. Gadovist^® ^- changes of signal intensities (ΔSi) over time

**Figure 11:**
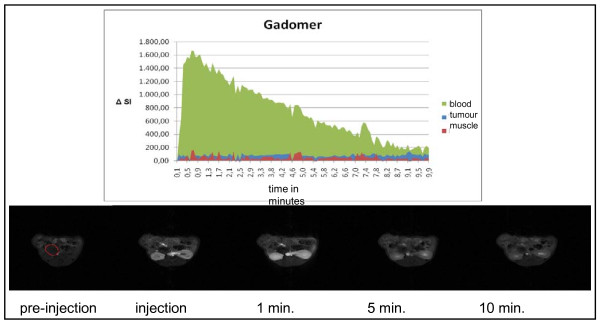
**Tumour blood flow analyses**. Gadomer^® ^- changes of signal intensities (ΔSi) over time

For these studies altogether n = 5 mice were analyzed. The low molecular weight contrast medium Gadovist^® ^as well as the high molecular weight contrast medium Gadomer^® ^resulted in an equivalent accumulation within the vascular system (Figure 10 and 11). As expected, Gadovist^®^, the low molecular weight contrast medium, showed higher accumulation within the tumour than Gadomer^®^.

For a better analysis of the tumour blood flow defined contrast-enhanced areas of the tumour, the muscles of the thigh, and the aorta were scanned. In these regions of interest (ROI) a quantitative comparison of the different signal intensities was carried out. These tests showed that an analysis of the tumour blood flow was possible and reproducible using either contrast medium. However, using the same concentrations of the contrast medium the two substances showed different signal intensities within the vascular system. Analysing the signal intensities within the aorta (ROI/mean) Gadomer^® ^showed values up to Δ1600 whereas Gadovist^® ^showed values only up to Δ650. Also, within muscle tissue and the tumour a significant difference was detectable. The low molecular weight Gadovist^® ^had a significantly increased accumulation within these tissues compared to the vascular system and therefore higher signal intensities whereas the high molecular weight Gadomer^® ^showed a significantly decreased accumulation implying lower signal intensities within muscle and tumour tissue compared to the vascular system.

For tumour blood flow analyses we found a concentration of the contrast medium of 0.0025 mmol/ml to be ideal. Using this concentration the analysis of the enhancement of the contrast medium employing VIBE sequences was optimal. At the same time the otherwise increasing possibility of biochemical interferences using higher concentrations of contrast media could be avoided.

## Discussion

*In vitro *and *in vivo *experiments are necessary to better understand the development and nature of malignant tumours. Furthermore, models that resemble the human situation are critical for developing and testing new therapeutic strategies. Here we used a 7 Tesla MRI for monitoring tumour growth and metastasis formation within liver and lymphnodes in a murine pancreatic cancer model. In addition, the blood flow within tumours could be analyzed. This model reliably mimics the clinical situation of diagnosing and monitoring pancreatic cancer patients by employing MRI [20,21].

The here utilized 7.0 Tesla small animal MRI scanner by Bruker (ClinScan, Bruker, Ettlingen, Germany) is part of a multidisciplinary core facility at Greifswald University. The basic work flow and work charts are similar to the already installed human MRI system which is controlled by Siemens software as is the small animal MRI. One new feature of the system, the possibility of generating breathing rate triggered sequences, has proved indispensable. The depth of anaesthesia controls nicely the breathing rate, enabling the investigator to keep the rate fairly stable. This is essential for breathing rate triggered sequences. Employing this technique, volumes of parenchymatous organs like the liver or the volume of solid tumours could be measured accurately down to differences in size as small as 0.1 mm. In the past, tumour growth and metastasis formation had been studied by direct measurement after sacrificing the animal. The MRI technique enables the researcher to monitor tumour development over time, if necessary several times, in the same animal. This has significantly reduced the number of required animals for longitudinal studies. At the same time, each mouse can serve as its own control comparing images at different time points. Also, for treatment experiments, tumours and their response to treatment can be easily monitored over time. The presented murine model in combination with the 7 Tesla MRI scanner is an excellent example of multidisciplinary cooperation. In this case radiologists, surgeons and physicians have shared their expertise to implement new and valuable tumour models to further improve the research of pancreatic cancer.

Many *in vivo *models have combined human cells and immunodeficient mice [8-11]. These models suffer from several short comings since normal tumour development and the role of the otherwise crucial immune system cannot be evaluated due to the impaired immune system. Also, human cells may behave differently once xenotransplanted into different species [9]. Therefore, syngeneic orthotopic tumour models in immunocompetent animals offer much more realistic conditions for studying malignancies and the impact on the immune system [12-15]. Modern techniques for studying the immune response, in particular methods to investigate cell trafficking, may employ modern MRI techniques [22-25].

Other tumour models have been introduced working simply with subcutaneously injected tumour cells [13,26]. The growth of these can be easily monitored by simple palpation or the use of a calliper. However, the microenvironment at the implantation site of the host organ can influence the natural history of tumour growth and the efficacy of antineoplastic agents. The microenvironment of an orthotopic model resembles that of naturally occurring tumours, and so experimental results in this model would be expected to have more relevance than results in a subcutaneous model [27]. However, for these models *in vivo *imaging systems are required.

The first reports on the use of MRI in small animals were published some 30 years ago [28]. The technique of MRI has steadily improved and has covered an increasing spectrum of applications. Nowadays, MRI technique provides a powerful tool for clinical imaging. Even for research applications it is broadly available, either as special small animal MR scanners or in combination with clinical devices. It offers good imaging performances and provides a tool for translational research [29].

The determination of sizes or volumes of implanted tumours without imaging techniques has employed the measurement using callipers or histology. In subcutaneous models calliper measurement is reasonable, since the mouse can be monitored over time without sacrificing it. In orthotopic tumour models this is not possible. Only after sacrificing the animal tumour sizes/volumes can be determined. Using callipers only approximations of the actual size or volumes of in situ tumours are possible. Tomayko et al. [19] have published extensively on mathematical formulas to correctly measure e.g. ellipsoid tumours. We have used the formula LxWxHxΠ/6 which has a correlation coefficient of 0.98 to 0.99. Even in larger or more irregularly shaped tumours the correlation is up to 0.92. Only for tumours larger than 17.0 g the coefficient will be r = 0.79, which is still acceptable. However, these formulas have been developed for subcutaneous tumours. MR avoids the necessity of sacrificing animals and irregular tumours can be measured more accurately.

Using histology we have found a very close correlation between histological size and size determined by MR. In fact, there was no difference in standard deviations. However, due to shrinking during fixation, changes during the processing of specimens including embedding, cutting and staining histological measurements were always somewhat smaller than MR measurements. The correctness of MR measurements were validated by algorithms developed by CIT and NIH specialists employing MIPAV software [17]. Furthermore, we confirmed our results by measuring phantoms with defined volumes with excellent recovery of the original volume.

The 7 Tesla small animal MRI scanner employed here has an excellent spatial resolution. Tumours were always readily detected. Using breathing controlled T2-weighted sequences tumour volumes could be measured very accurately. We could find a plane image resolution of 0.17 mm × 0.13 mm when using slices of 0.7 mm. Due to this high resolution MRI was highly suggestive of tumour infiltration of surrounding organs if invasion was present. When MRI was inconclusive histological examination could differentiate tumour infiltration from inflammation [30]. In fact, the same is true for the human situation. Working with these models over time including the combination of clinical, macroscopic, microscopic and MR images had a significant training effect and improved the accuracy of interpreting difficult results or lesions of the images.

Several *in vivo *imaging systems other than MRI, such as CT, CT-PET, ultrasound, bioluminescence and fluorescence imaging techniques, have been developed for determining tumour size and volume in orthotopic experimental models [31]. These techniques have dramatically reduced the number of required animals according to the 3R principles described by Russel and Burch 1959 [16]: each animal can be scanned repeatedly in a longitudinal study, avoiding the need for large control groups. However, the MRI technique combines several advantages making it the preferred imaging technique. MRI offers better discrimination of soft tissues and local resolution [32]. Also, MRI offers a broader spectrum of dynamically contrast enhanced scanning options and can, in contrast to fluorescence techniques, image the complete animal [33]. Also, the experimental set-up as well as MRI-technique itself allows imaging using contrast media, scanning the animal for longer periods and allowing repeated imaging series. Finally, MRI does not employ any radiation and, other than ultrasound, is less investigator-dependent [34].

At the beginning of small animal MRI scanning many problems had been caused by moving artefacts due to difficulties in anaesthesia, the breathing of animals and bowel motility. Modern techniques and the use of the 7 Tesla MRI scanner have greatly reduced these problems [29]. The inhalative anaesthetic isoflurane and its application via an adjustable evaporation system has shown many advantages compared to intraperitoneal anaesthesia using agents such as ketamine hydrochloride. The amount of administered isoflurane is easily adjusted allowing a reliable and stable control of breathing rates. Despite its moderate cardiodepressive actions it is well tolerated by animals for long imaging series of up to 60 minutes [35]. Furthermore, ketamine or xylazine may have dramatic effects on the metabolism of small animals, in particular glucose metabolism, having a significant impact on functional studies [36]. The reliable and stable control of the breathing rate was required for breathing rate triggered sequences. This led to reduced artefacts and allowed exact measuring of tumour volumes using T2-weighted sequences [37]. For similar purposes the reduction of bowel motility by starving the animals for 4 hours prior to MR imaging is a helpful tool to reduce motion artefacts of the bowel. A further reduction of bowel motility by N-butyl-scopolamine was not required. Its short half-life of 5-10 minutes and its possibly hazardous side effects might jeopardize MRI analyses. Grimm et al. have published similar findings concerning MR scanning of the pancreas [38]. All these aforementioned supporting techniques improved the focus, leading to a better visualization of the tumour margins and detection of tumour invasion into surrounding tissues. The injection of contrast media was not necessary for these analyses.

For MRI scans of the abdomen the whole body mouse coil generated the best images. In contrast to conventional surface coils it is a volume coil and has a very high spatial resolution although a slightly reduced signal to noise ratio. Conventional surface coils like the rat brain coil (2 × 2), which could also be used, showed decreased signal intensities with increased coil distance. The whole body mouse coil avoided inhomogeneities of the magnetic fields. This guarantees the continuous good quality of images of the whole FoV including a good signal to noise ratio.

For the induction of liver metastases several models including the portal vein injection technique [39] and the direct microinjection model [40] have been published. We followed the suggestions of Ishizu et al. [41] who established the splenic injection model. It is the most efficient model and has the lowest rate of complications i.e. post surgical bleeding and local tumour spread. So far, all animals have formed liver metastases of the injected pancreatic cancer cell lines. With the help of the liver specific contrast medium Primovist^®^, comparable to the human situation, metastatic lesions could be accurately detected with a significantly improved focus of the lesions. Using the MRI software MIPAV this is an ideal model for comparative and therapeutic studies involving either liver metastases alone or liver metastases and their primary tumour. The amount and size of liver lesions and, if present, the primary tumour, can be exactly monitored over time without sacrificing animals, again complying with the 3R principles [16].

In this study we have routinely used the intravenous contrast medium Primovist^® ^for the detection of liver metastases and the intravenous contrast media Gadovist^® ^and Gadomer^® ^for tumour blood flow studies employing an orthotopic murine pancreatic cancer model. These models very closely resemble the human situation. Interestingly, He et al. [42] described problems with the intravenous use of contrast media whereas Grimm et al. suggested the intraperitoneal application of contrast media [38].

The low molecular weight contrast medium Gadovist^® ^as well as the high molecular weight contrast medium Gadomer^® ^can be used for dynamic contrast enhanced imaging of the tumour blood flow and for analyzing different factors that might influence tumour blood flow.

Gadovist^® ^shows a much higher accumulation of contrast medium within the parenchyma of the tumour than the high molecular weight Gadomer^®^. However, high molecular weight contrast media could be used to study tumour neoangiogenesis [35]. VEGF as the critical cytokine in tumour neoangiogenesis could be therapeutically blocked by inhibitors such as anti-VEGF-antibodies or anti-VEGF-receptor-antibodies or other therapeutic agents. Since VEGF could lead to an increased permeability of tumour capillaries Gadomer^® ^could show an increased accumulation in areas of increased VEGF activity and a decreased accumulation in areas of decreased or blocked activity. In those studies the dynamic contrast enhanced MRI would demonstrate direct therapeutic effects. Also, they could be monitored over time, again significantly reducing the number of research animals as asked for by Russel and Burch [16].

## Conclusions

The analysis of murine pancreatic cancer models can be done comparably to the human situation. Employing a 7 Tesla small animal MRI in a multidisciplinary environment involving radiologists, physicians and surgeons, allows the exact clinical detection and monitoring of tumour growth, tumour volumes, lymphnode and liver metastasis formation as well as the tumour blood flow. The number of research animals can be significantly reduced in longitudinal studies according to the 3R principles. The presented models can be used for imaging studies as well as non-surgical and surgical treatment purposes.

## Competing interests

The authors declare that they have no competing interests.

## Authors' contributions

LIP worked on the study conception and its design and participated in all experiments, statistics and drafting and revision of the manuscript. He read and approved the final manuscript. AK also worked on the study conception and its design and participated in all MR and mouse experiments including surgery. He was involved with statistics, the revision of the manuscript and read and approved the final manuscript. MS performed MR and mouse experiments including surgery. He was involved with interpretation of data, revision of the manuscript as well as reading and approval of the final manuscript. NA carried out MR, histology and mouse experiments including surgery. She took part in the interpretation of data, the revision of the manuscript and read and approved its final version. JPK worked on the study design and MR experiments as well as the interpretation of data and the revision of the manuscript. He read and approved the final manuscript. SS took part in the study design and MR and histology experiments. He interpreted the data and helped with statistics and revision of the manuscript. He read and approved the final manuscript. SR participated in the study design, histology experiments, statistics, the interpretation of data as well as revision of the manuscript. He read and approved its final version. FS also participated in the study design, histology experiments, statistics, the interpretation of data and the revision of the manuscript. He read and approved the final manuscript. SD was involved in MR experiments, the revision of the manuscript and read and approved the final manuscript. SK collected MR and calliper data, performed mouse experiments including surgery and was involved with statistics, the interpretation of data and the revision of the manuscript. She read and approved the final manuscript. FUW participated in the study conception, the interpretation of data and revision of the manuscript. He read and approved the final manuscript. JM worked on the interpretation of data, the revision of the manuscript and read and approved its final version. MML participated in the interpretation of data, the revision of the manuscript and read and approved the final manuscript. SH worked on MR experiments, the interpretation of data and the revision of the manuscript. He read and approved the final manuscript. NH was involved with the interpretation of data, the revision of the manuscript and read and approved its final version. CDH interpreted the data, revised the manuscript and read and approved the final manuscript. RP was also involved with the interpretation of data and the revision of the manuscript. He read and approved the final manuscript. WvB also worked on the study conception and its design. He performed statistics, interpreted the data and was involved with drafting and revising the manuscript. He read and approved the final manuscript.

## Pre-publication history

The pre-publication history for this paper can be accessed here:

http://www.biomedcentral.com/1471-2407/11/40/prepub
